# Specific changes in circulating cytokines and growth factors induced by exercise stress testing in asymptomatic aortic valve stenosis

**DOI:** 10.1371/journal.pone.0173787

**Published:** 2017-03-14

**Authors:** Renata Kolasa-Trela, Malgorzata Konieczynska, Marta Bazanek, Anetta Undas

**Affiliations:** 1 Department of Diagnostic Medicine, John Paul II Hospital, Krakow, Poland; 2 Institute of Cardiology, Jagiellonian University Medical College, Krakow, Poland; Centro Cardiologico Monzino, ITALY

## Abstract

**Background:**

We evaluated exercise-induced changes in the profile of circulating cytokines and growth factors in patients with AS.

**Methods:**

We studied 32 consecutive asymptomatic moderate-to-severe AS patients and 32 age and sex-matched controls. Plasma levels of interleukin (IL)-6, IL-10, hepatocyte growth factor (HGF), vascular endothelial growth factor (VEGF), and transforming growth factor (TGF)-β were measured at 4 time points, i.e. at rest, at peak bicycle exercise, one hour and 24 hours after a symptom-limited exercise.

**Results:**

Exercise increased all the 5 markers in both groups (all p<0.0001). The maximum levels of all tested cytokines were higher in the AS group (all p<0.05) compared with controls. In AS patients the highest levels of VEGF, IL-6, and IL-10 were observed one hour after exercise, while in the control group at peak exercise. In both groups maximum TGF- β levels were observed one hour after exercise. HGF levels were higher at peak and one hour after test in the AS group (p = 0.0001), however the maximum value in AS was observed at peak while in controls after test. In both groups TGF-β was the only marker that remained increased 24 hours after exercise compared with the value at rest (p = 0.0001). The cytokines and growth factors showed no association with heart rate and the workload.

**Conclusion:**

In asymptomatic patients with moderate-to-severe AS, exercise produces a different pattern of changes in circulating cytokines and growth factors, and maximum levels of all tested cytokines were significantly higher in AS patients compared with the control group.

## Introduction

Aortic valve stenosis (AS), the most common valvular heart disease in adults, is an active process with accumulation of lipids and inflammatory cells, extracellular matrix remodeling, angiogenesis and calcium deposition, which is to some extent similar to atherosclerosis [1].

AS is characterized by an abnormal mineralization linked with inflammation and neovascularization [2,3]. A combination of endothelial damage and lipid deposition triggers inflammation within the valve. The infiltration of the endothelial layer predominantly composed of macrophages and lymphocytes that release pro-inflammatory cytokines stimulate and establish fibrotic and calcific processes that drive increasing valve stiffness [4]. Inflammatory cells produce several cytokines that can promote the activation and differentiation of vascular interstitial cells (VICs) [5,6]. Previous studies demonstrated the increased expression of cytokines including interleukin (IL)-1, IL-6, and transforming growth factor (TGF)-β in calcified human aortic valves [7]. Recently, El Hussseini et al. have shown that IL-6 is an important mediator of mineralization and is secreted by VICs in response to phosphate [8]. It has been demonstrated that TGF-β is expressed within calcific aortic cusps, induces calcification and collagen synthesis in cultured valvular VICs [9,10]. Stenotic aortic valves are vascularized and it contributes the progression of disease by facilitating the entry of inflammatory cells into the leaflets [2, 11–14]. Other growth factors like vascular endothelial growth factor (VEGF)-C and VEGF-D which promote angiogenesis are locally produced by endothelial cells and myofibroblasts of aortic valve leaflets [15]. Hepatocyte growth factor (HGF), a mesenchymal derived factor, has been regarded as a intermediator of epithelial-mesenchymal interplays and a powerful inducer of angiogenesis [16,17]. Recent studies have suggested that HGF had a marked effect on the tissue repair by suppression of proinflammatory cytokine production [17,18]. Endothelium is extremely sensitive to changes in the blood flow, so turbulence at the supravalvular region in AS, that increases during exercise, can cause endothelial cell injury followed by a local inflammation. On the other hand, it is known that exercise protects against disease associates with chronic low-grade systemic inflammation [19]. Beneficial effects of physical activity in terms of lower risk of coronary artery disease (CAD) and cardiovascular mortality may result at least in part from modulation of inflammation following exercise. Konstantinos et al. demonstrated that exercise performed in CAD patients is safe without exacerbating underlying inflammation. They observed that exercise elicited normal pro-and anti-inflammatory responses, analyzing concentrations of tumor necrosis factor (TNF)-α, high sensitivity C-reactive protein (hs-CRP), interferon (INF)-γ, IL-6, IL-10 and TGF-β [20].

To our knowledge, there have been no studies investigating the effect of exercise on circulating concentrations of cytokines and growth factors in AS patients as compared to well-matched controls. We hypothesized that exercise-induced hemodynamic changes unfavorably alter the profile of circulating cytokines and growth factors in AS patients as compared to well matched healthy controls.

## Materials and methods

### Study design

We enrolled 33 patients (16 males and 17 females) with asymptomatic moderate-to-severe AS defined as transvalvular maximal velocity [V_max_] ≥3 m/s [21]. Sex- and age-matched individuals recruited from the hospital staff and relatives served as controls. The two groups have been described in detail previously [22]. Briefly, the exclusion criteria were: history of angina, myocardial infarction, stroke, dizziness, or syncope, another cardiac valve disease of more than a moderate degree, left ventricular (LV) ejection fraction (EF) <50%, prior or current atrial fibrillation, hyper- or hypothyroidism, diabetes treated with insulin, renal or liver dysfunction, chronic pulmonary disease, treatment with oral anticoagulants, thienopyridine or nonsteroidal anti-inflammatory drugs other than aspirin, known cancer, autoimmune disorders, inability to perform exercise testing. The study protocol was approved by the Bioethics Committee of the District Medical Chamber, and each patient provided written, informed consent to participate in the study.

To evaluate the extent of atherosclerotic vascular disease, which coexists in about 50% of AS patients [23], we measured intima-media thickness (IMT) in both right and left common carotid artery in accordance with the Mannheim IMT consensus [24]. Ankle Brachial Pressure Index (ABI) was measured using an arterial pressure sphygmomanometer and a continuous wave Doppler ultrasound blood flow detector and the ABI values of 0.9–1.15 were considered normal. Hypertension was diagnosed based on a history of arterial hypertension or antihypertensive treatment. Hyperlipidaemia was diagnosed based on medical records, statin therapy or total cholesterol of ≥5.0 mmol/L.

### Echocardiography

Transthoracic echocardiography was performed in all subjects using Philips iE33 device.

LV volumes and EF were measured by the biplane Simpson’s method. The aortic valve area (AVA) was calculated using the standard continuity equation. V_max_, peak pressure gradient (PPG) and mean pressure gradient (MPG) were calculated using the modified Bernoulli equation.

A symptom-limited exercise stress echocardiography was performed on a bicycle ergometer (Ergoline) in a semi supine position with a continuous echocardiographic examination by an experienced cardiologist. After 3 minutes of the initial workload of 25W, the workload was increased every 3 minutes by 25W. ECG was monitored and blood pressure was measured every 3 minutes during exercise. Exercise was stopped in case of typical chest pain, breathlessness, dizziness, muscular exhaustion, hypotension, ventricular arrhythmia, when age-related maximum heart rate was reached or on patient’s demand. The test was performed at rest and at peak exercise.

### Laboratory tests

Fasting blood samples were drawn from the antecubital vein between 7 and 10 a.m. Fibrinogen was measured by the von Clauss method. High-sensitivity C-reactive protein was determined using immunoturbidimetry (Roche Diagnostics, Mannheim, Germany). Blood samples were drawn four times: at rest, at peak exercise, one hour and 24 hours after exercise. Blood was centrifuged at 2500 g at 20°C for 10 min and stored at -80°C until analysis. Technicians were blinded to the origin of the samples.

Plasma levels of the following biomarkers: IL-6 and IL-10, HGF, VEGF, and TGF-β were measured with the use of commercially available enzyme-linked immunosorbent assays (ELISA; R&D Systems, Abington, UK) according to the manufacturer’s instructions.

### Statistical analysis

Statistical analysis was performed using STATISTICA 10 PL software package. Values are presented as a mean± standard deviation or median or otherwise stated. The Shapiro-Wilk test was performed to determine normal distribution of the variables. The Student’s t test was used to determine differences among normally distributed variables and the Mann–Whitney U test for non-normally distributed variables. Serial tests were analyzed using Friedman ranks analysis of variance. A linear Pearson correlation was used to assess correlations among variables. A two-sided p-value <0.05 was considered statistically significant.

## Results

Thirty-two AS patients (MPG of 35.4±14.1 mmHg, AVA = 1.08±0.23 cm2;) and 32 controls were included in the final analysis (Table 1).

**Table 1:** Baseline characteristics.

Variables	AS patients (n = 32)	Controls (n = 32)	p
Males, n (%)	15 (46.8)	17 (53.1)	0.62
Age (years)	64 (23–82)	63.5 (33–83)	0.81
BMI (kg/m^2^)	30 (21–37)	28 (21–39)	0.19
Current Smoking, n (%)	2 (6.2)	2 (6.2)	1.0
Hypertension, n (%)	26 (81.2)	27 (84.3)	0.74
Diabetes, n (%)	7 (21.8)	4 (12.5)	0.32
Hypercholesterolaemia, n (%)	12 (37.5)	2 (6.2)	0.02
***Laboratory investigations***			
Fibrinogen (g/L)	3.26 ± 0.7	3.04 ± 0.5	0.21
Total cholesterol (mM)	5.03 ± 1.04	5.26 ± 1.2	0.41
LDL cholesterol (mM)	3.04 ± 0.8	3.33 ± 1.0	0.24
HDL cholesterol (mM)	1.48 ± 0.3	1.48 ± 0.4	0.92
Triglycerides (mM)	1.15 (0.4–4.2)	1.15 (0.4–2.3)	0.50
Glucose (mM)	5.85 ± 1.1	5.38 ± 0.5	0.30
Creatinine (μM)	73.8 ± 12.4	77.3 ± 13.9	0.28
C-reactive protein (mg/L)	1.23 (0.3–10.4)	1.6 (0.2–8.0)	0.61
***Treatment***			
Aspirin, n (%)	18 (56.2)	10 (31.2)	0.044
Statins, n (%)	23 (71.8)	20 (62.5)	0.42
β-blockers, n (%)	19 (59.3)	12 (37.5)	0.08
ACEI, n (%)	12 (37.5)	15 (46.8)	0.45

Data are median (interquartile range) or mean ± SD, unless otherwise stated. P value was measured using Student t-test when variables were normally distributed or by the Mann-Whitney U-test for non-normally distributed variables. Abbreviations: BMI, body mass index; LDL, low-density lipoprotein; HDL, high-density lipoprotein; ACEI, angiotensin-converting enzyme inhibitor

The duration of stress test was shorter in AS group (9.0±1.9 min vs 10.8±2.7 min; p = 0.008) and maximum workload was lower (81.3±21.1 W; p = 0.002). Echocardiographic data are presented in Table 2.

**Table 2:** Clinical, exercise and echocardiographic data.

Variables	AS (n = 32)	K (n = 32)	p
**Clinical and Exercise Data**			
***Resting***			
Heart rate (bpm)	65.3 ± 10.1	68.1 ± 10.9	0.423
Systolic arterial pressure (mm Hg)	131.9 ± 10.1	133.6 ± 11.9	0.870
Diastolic arterial pressure (mm Hg)	76.8 ± 8.8	78.6 ± 6.9	0.549
Maximal workload (WATT)	81.3 ± 21.1	100.8 ± 22.4	0.002
Duration (min)	9.0 ± 1.9	10.8 ± 2.7	0.008
***Exercise***			
Heart rate (bpm)	113.3 ± 21.9	122.7 ± 17.4	0.043
Systolic arterial pressure (mm Hg)	158.4 ± 10.2	166.5 ± 18.3	0.108
Diastolic arterial pressure (mm Hg)	83.1 ± 5.6	84.9 ± 9.1	0.257
**Resting and Exercise Echocardiographic Data**			
***Resting***			
LVEF (%)	66.3 ± 7.3	69.8 ± 7.6	0.079
LVEDV (ml)	96.4 ± 27.7	92.7 ± 28.3	0.519
LVEDV/BSA (ml/m^2^)	51.9 ± 13.4	49.2 ± 13.7	0.376
LV mass (g)	208.8 ± 59.6	150.7 ± 43.1	<0.001
LV mass BSA (g/m^2^)	109.5 ± 24.0	78.4 ± 20.1	<0.001
LA area (cm^2^)	20.3 ± 4.7	15.5 ± 2.9	<0.001
LA volume (ml)	58.5 ± 23.1	40.5 ± 11.5	<0.001
LA volume//BSA (ml/m^2^)	29.9 (17.6–65)	22.5 (8.3–35)	<0.001
Ascending aorta (mm)	38.7 ± 5.7	32.8 ± 3.9	<0.001
PPG (mmHg)	59.4 ± 19.8	8.0 ± 3.6	<0.001
MPG (mmHg)	35.4 ± 14.1	4.4 ± 1.8	<0.001
AVA (cm^2^)	1.0 ± 0.23		
AVA/BSA (cm^2^/m^2^)	0.58 ± 0.11		
***Exercise***			
PPG (mmHg)	75.3 ± 26.1		
MPG (mmHg)	45.3 ± 15.9		
AVA (cm^2^)	1.1 ± 0.3		
AVA/BSA (cm^2^/m^2^)	0.58 ± 0.14		

Abbreviations: LVEF- left ventricular ejection fraction; LVEDV- left ventricular end diastolic volume; BSA- body surface area; LV- left ventricle; LA- left atrium; PPG- peak pressure gradient; MPG- mean pressure gradient; AVA- aortic valve area

### Interleukin-6

**Fig 1:**
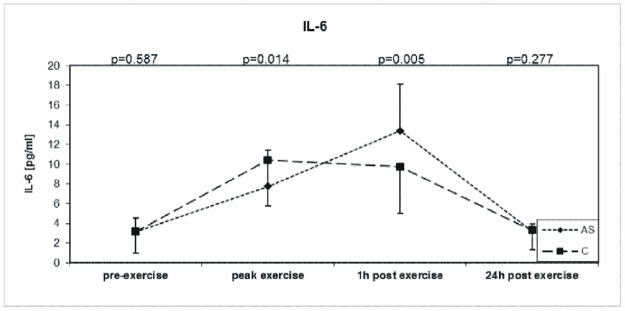
Exercise-induces changes during symptom-limited exercise on a bicycle ergometer in 32 asymptomatic aortic valve stenosis patients (AS) and 32 well-matched Controls (C) in Interleukin- 6 (IL-6).

The baseline IL-6 concentrations were similar in the AS and control groups (Fig 1 and Table 2). In response to exercise IL-6 levels increased (+325%) with the highest levels one hour after exercise in AS patients, while IL-6 rose similarly to maximum value at peak exercise (+231%) in the control group (Table 2). In the AS group we found positive correlations between baseline IL-6 and BMI (r = 0.693, p = 0001). The level of IL-6 measured 1h post exercise correlated with total cholesterol (r = 0.378, p = 0.033) and LDL cholesterol (r = 0.366, p = 0.040). The level of IL-6 measured 24 h post exercise correlated with age (r = 0.518, p = 0.02) and with hs-CRP (r = 0.343, p = 0.054).

### Interleukin-10

**Fig 2:**
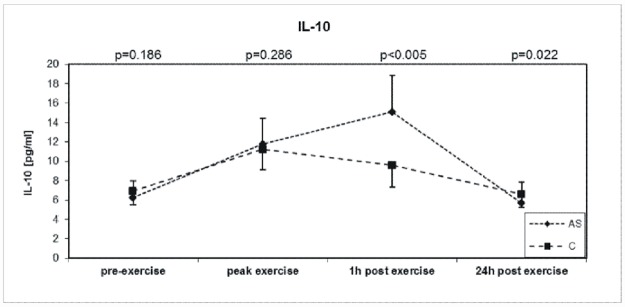
Exercise-induces changes during symptom-limited exercise on a bicycle ergometer in 32 asymptomatic aortic valve stenosis patients (AS) and 32 well-matched Controls (C) in Interleukin- 10 (IL-10).

IL-10 was similar at baseline and at peak exercise in both groups, but one hour after exercise it was higher (+141%) in AS group and (+61.8%) in the control group (Table 2, Fig 2).

### TGF-β

**Fig 3:**
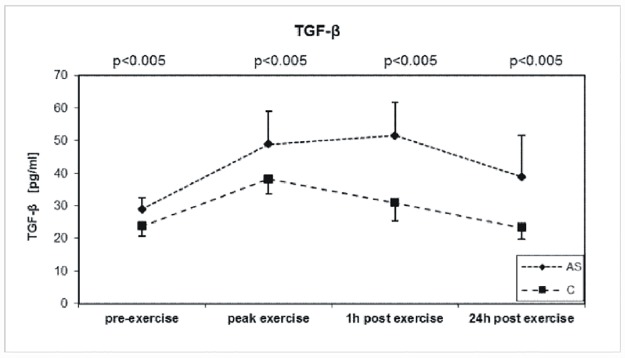
Exercise-induces changes during symptom-limited exercise on a bicycle ergometer in 32 asymptomatic aortic valve stenosis patients (AS) and 32 well-matched Controls (C) in TGF-β (transforming growth factor).

As shown in Fig 3 and Table 2, the levels of TGF-β were higher among AS patients than in the control group both at baseline, peak exercise, one and 24 hours after exercise (all p<0.005). In AS group TGF-β was increased during the test with the highest levels one hour after exercise (+77.7%). In the control group the highest TGF-β levels were observed at peak exercise (+60.4%). In AS patients TGF-β detected at baseline and 24 h post exercise correlated with hs-CRP (r = 0.44, p = 0.011; r = 0.561, p = 0.01, respectively), but not with fibrinogen or demographics.

### HGF

**Fig 4:**
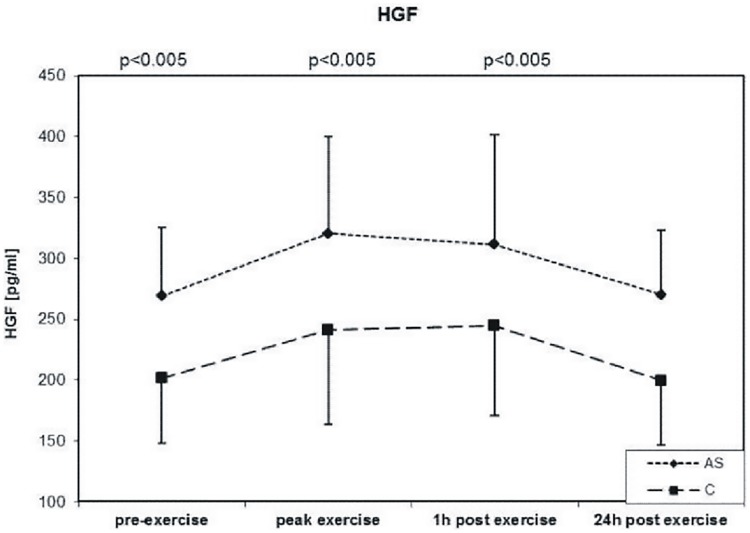
Exercise-induces changes during symptom-limited exercise on a bicycle ergometer in 32 asymptomatic aortic valve stenosis patients (AS) and 32 well-matched Controls (C) in HGF (hepatocyte growth factor).

In response to exercise HGF levels increased in both groups with the highest levels at peak exercise. In AS patients the levels of HGF were higher in all time points compared with the controls (+21%), (all p< 0.005) (Fig 4, Table 2). In AS group age correlated with HGF at baseline (r = 0.351, p = 0.049), 1 h (r = 0.361, p = 0.042) and 24h post exercise (r = 0.469, p = 0.007). HGF showed no association with inflammatory markers or lipid variables (data not shown).

### VEGF

**Fig 5:**
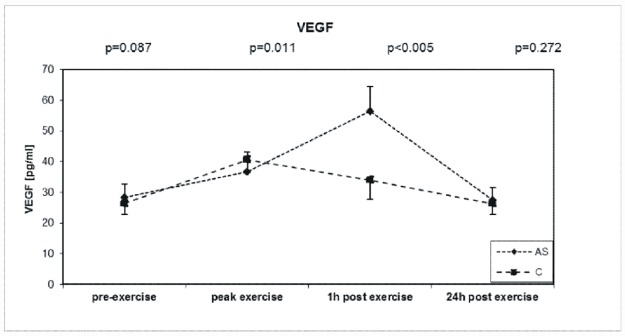
Exercise-induces changes during symptom-limited exercise on a bicycle ergometer in 32 asymptomatic aortic valve stenosis patients (AS) and 32 well-matched Controls (C) in VEGF (vascular endothelial growth factor).

The baseline level of VEGF was similar between AS patients and the control group. Exercise induce the highest level of VEGF was observed one hour after exercise in AS (+100%) and was higher compared with the controls (+54.1%) (Fig 5). In AS group baseline VEGF and 24h post exercise test levels correlated with BMI (r = 0.802, p = 0.001; r = 0.684, p = 0.001, respectively).

#### Associations with echocardiographic variables

In both groups we did not observe any associations of the biomarkers tested with heart rate, the workload calculated in Watts. Of the studied parameters describing the severity of AS, we found the only positive correlation between AVA and IL-6 levels at rest (r = 0.426, p = 0.015).

## Discussion

The current study is the first to demonstrate that exercise modulates the release of cytokines and growth factors in asymptomatic patients with moderate-to-severe AS and the profile of exercise-induced changes significantly differs in AS from that observed in well-matched controls. The same cohort was also investigated how stress echocardiography alters blood coagulation and fibrinolysis, as described in detail previously [22]. At rest AS patients had higher HGF and TGF-β but not IL-6, IL-10 and VEGF levels. Our study showed that the post-exercise increases in plasma concentrations of IL-6, IL-10, TGF-β, HGF and VEGF were more pronounced and maximum levels of all tested cytokines were significantly higher in AS patients compared with the controls. Interestingly, the peak value of Il-6, IL-10, TGF-β and VEGF was observed one hour post-exercise. This study increases our knowledge on a complex role of exercise in modulation of cytokine balance and indicates that hemodynamically significant heart valve defects may alter the profile of circulating cytokines and growth factors with the largest impact on IL-6.

### IL-6

It is known that IL-6 is abundantly released into the circulation during exercise [19].

It has been reported that in healthy, young volunteers IL-6 levels were significantly elevated immediately post-exercise [25]. The gene expression of IL-6 in skeletal muscle increases following exercise [26] and the main source of the release of IL-6 were skeletal muscle [27]. Adipose tissue and peripheral blood mononuclear cells may also contribute to IL-6 in the circulation at rest [28]. Data on post-exercise changes of IL-6 levels in atherosclerosis related diseases are equivocal. In patients with chronic heart failure, a nonsignificant increase of IL-6 following exercise has been shown [29]. Volaklis et al. observed an increase in IL-6 levels immediately after exercise in middle-aged patients with CAD [30]. Previous studies demonstrated elevated IL-6 levels in AS patients [31]. Expression of IL-6 on stenotic aortic valve leaflets was also observed [7,8]. We provided evidence that in AS group, IL-6 levels markedly increased during exercise and the maximum levels were significantly higher as compared to the control. It can be concluded that AS patients have increased pro-inflammatory burden compared to the healthy population and they have stronger response to the exercise challenge. A positive correlation of exercise-induced increase in IL-6 with AVA in the present study suggests a close link between the severity of the disease and IL-6-mediated inflammatory state.

### IL-10

IL-10 is a cytokine with pleiotropic effects affecting immunoregulation and inflammation. Recently it has been reported that genetic polymorphism of IL-10 may be associated with the susceptibility to valvular calcification [32].

The studies carried out in healthy young volunteers indicate that there is a dissociation between exercise-induced local gene expression in skeletal muscle and systemic concentration of IL-10 [26]. Conversely, the circulating concentration of IL-10 increases markedly, but this cytokine is not expressed in skeletal muscle after exercise [26].

Among patients with AS we observed a significantly higher and prolonged post exercise increase of IL-10 compared to the control group. Given the stimulating effect of exercise on the release of pro-inflammatory cytokines including TNF-alpha and the inhibitory effect of IL-10 on TNF—alpha [33], the increase in serum IL-10 levels in the AS group can be considered a compensative anti-inflammatory response.

### TGF-β

TGF-β is a multifunctional cytokine which has proliferative activity on cardiac and valvular fibroblasts. TGF-β induces hypertrophy and apoptotic cell death in cardiomyocytes and with the other cytokines it ultimately stimulates fibrotic and calcific processes that drive valve stiffness [4]. The presence of TGF-β has been reported in stenotic aortic valves [7].

Peake et al. have reported that plasma concentrations of TGF-β remained unchanged following exercise [26]. However, Volaklis et al. observed an increase of TGF-β only in CAD patients in whom the low intensity protocol was used [30]. We observed increased TGF-β not only 1 hour after exercise but even 24 hours after exercise in AS patients. Valvular endothelial cells respond to local shear stress changes to modulate intracellular signaling which leads to altered gene expression, cell morphology and structural remodeling [34]. Healthy aortic valve endothelium is resistant to molecular diffusion and cell penetration into the tissue interstitial space and bloodstream. Leaflets exposed to altered shear stress demonstrate increased expression of the inflammatory proteins, TGF- β, only on the aortic side that indicates the side-dependent shear sensitivity [35]. In animal models of AS, it has been demonstrated that there is a relationship between shear stress and serum TGF-β levels [36]. An exercise by increasing shear stress [37] and turbulent blood flow in the supravalvular region can activate TGF-β in the aortic valve endothelial cells [35], which may explain a higher and prolonged post-exercise increase of serum TGF-β observed in the AS group.

### HGF

HGF counteracts the activity of TGF-β. HGF suppresses myocardial hypertrophy and its down-regulation activity on fibrogenic and hypertrophic genes is associated with improved cardiac function. HGF enhances endothelial NO production [38]. It was reported that HGF increase induced by pharmacological stimulation could favorably improve exercise-induced ischaemia in patients with CAD [39]. Wahl et al. demonstrated post-exercise increase in HGF (3 hours after) in young, healthy non-smoking males [16]. We observed higher levels of HGF both at baseline and post exercise in the AS group. It might be speculated that in AS, the post-exercise HGF release is protective by inhibition of apoptosis and enhancement of valvular endothelium repair.

### VEGF

VEGF has been demonstrated in stenotic aortic valves, precisely [15,40]. VEGF act predominantly on vascular endothelial cells by stimulating neoangiogenesis and facilitating the entry of inflammatory cells and lipids into the leaflets, thus accelerating progression of AS [14]. Increased plasma VEGF in aged patients associates with AS [41]. Wahl et al. demonstrated an immediate post-exercise increase in VEGF levels in young, healthy non-smoking males [16]. Kraus et al. also observed an increase in VEGF immediately after and 2 hours after exercise in well-trained athletes [42]. Our findings showed that in AS the maximum levels of VEGF were markedly higher compared with the controls and was observed one hour after exercise. Based on our findings, it might be hypothesized that the post-exercise increase in angiogenic factors (VEGF and TGF-β) in the AS group may affect remodeling of the stenotic aortic valve.

### The relationship between exercise and cytokine release

Previous observations indicate that not all of cytokines expressed in skeletal muscles are released during post- exercise period into the circulation [26].

Importantly the duration of bicycle exercise was shorter and maximum workload was lower in the AS group compared to the control but the maximum levels of cytokines were higher, which further suggest other than muscle source of its release.

In our opinion the cytokine release in AS was induced by exercise and this effect was enhanced by chronic low-grade inflammation, therefore differed markedly from controls.

It is likely that the release of cytokines beyond the known source of the muscle occurs locally in the supravalvular region. Turbulence at supravalvular region in AS patients, that increases during exercise, can damage the endothelium followed by a local pro-inflammatory state. It is known that exercise protects against diseases associated with chronic low-grade systemic inflammation [24], however it is unclear whether post-exercise increase of cytokines observed in AS is protective. Exercise-induced increases in blood flow and shear stress have been observed to enhance vascular function and structure. Where there is a laminar flow, the adhesion of inflammatory molecules and activation of pro-inflammatory cascade are reduced. By increasing the release of nitric oxide and prostacyclin, shear stress augments endothelium-dependent vasodilatation and inhibits multiple processes involved in atherogenesis [43].

In AS the blood flow in the supravalvular region is turbulent compounded by the effort.

It is unknown whether exertional increased activity of cytokines may slow down the inflammatory process ongoing on the valve and inhibit its remodeling or whether it is only an indicator of hemodynamic disruption. It is still controversial whether inflammatory mediators have primarily causal or counter-regulatory functions [44]. On the other hand, since activated cytokines can be a stimulus and result of valvular calcification alike (vicious cycle by which inflammation promotes VICs and VICs itself promotes inflammation) [45,46]. Kastellanos et al. have clearly showed that the decrease in the levels of inflammatory cytokines after aortic valve replacement, however before aortic valve replacement there was no relation between inflammatory markers and echocardiographic parameters [47]. We suggest that the exercise-stimulated increase of pro-inflammatory cytokines is undesirable.

### Study limitations

Both study groups were small, however their size was sufficient to show the intergroup differences in measured parameters. We measured only serum levels of cytokines, gene expression was not identified. Our findings cannot be likely extrapolated to the whole population of AS patients since the study participants with mild or symptomatic AS were excluded.

### Conclusion

Our study demonstrated that in asymptomatic patients with moderate-to-severe AS, exercise stimulate a higher increase in pro-inflammatory cytokines than that in the control group, suggesting enhanced post-exercise inflammatory state in AS.

## Supporting information

S1 File Raw study data. (XLSX)
